# Hopping into a hot seat: Role of DNA structural features on IS*5*-mediated gene activation and inactivation under stress

**DOI:** 10.1371/journal.pone.0180156

**Published:** 2017-06-30

**Authors:** M. Zafri Humayun, Zhongge Zhang, Anna M. Butcher, Aref Moshayedi, Milton H. Saier

**Affiliations:** 1 Department of Microbiology, Biochemistry & Molecular Genetics, Rutgers—New Jersey Medical School, Newark, NJ, United States of America; 2 Department of Molecular Biology, Division of Biological Sciences, University of California San Diego, La Jolla, CA, United States of America; University of Helsinki, FINLAND

## Abstract

Insertion sequence elements (IS elements) are proposed to play major roles in shaping the genetic and phenotypic landscapes of prokaryotic cells. Recent evidence has raised the possibility that environmental stress conditions increase IS hopping into new sites, and often such hopping has the phenotypic effect of relieving the stress. Although stress-induced targeted mutations have been reported for a number of *E*. *coli* genes, the *glpFK* (glycerol utilization) and the cryptic *bglGFB* (β-glucoside utilization) systems are among the best characterized where the effects of IS insertion-mediated gene activation are well-characterized at the molecular level. In the *glpFK* system, starvation of cells incapable of utilizing glycerol leads to an IS*5* insertion event that activates the *glpFK* operon, and enables glycerol utilization. In the case of the cryptic *bglGFB* operon, insertion of IS*5* (and other IS elements) into a specific region in the *bglG* upstream sequence has the effect of activating the operon in both growing cells, and in starving cells. However, a major unanswered question in the *glpFK* system, the *bgl* system, as well as other examples, has been why the insertion events are promoted at specific locations, and how the specific stress condition (glycerol starvation for example) can be mechanistically linked to enhanced insertion at a specific locus. In this paper, we show that a specific DNA structural feature (superhelical stress-induced duplex destabilization, SIDD) is associated with “stress-induced” IS5 insertion in the *glpFK*, *bglGFB*, *flhDC*, *fucAO* and *nfsB* systems. We propose a speculative mechanistic model that links specific environmental conditions to the unmasking of an insertional hotspot in the *glpFK* system. We demonstrate that experimentally altering the predicted stability of a SIDD element in the *nfsB* gene significantly impacts IS*5* insertion at its hotspot.

## Introduction

Transposable genetic elements, once considered to be exotic “DNA level parasites” that enabled non-Mendelian transmission of genetic traits, are now recognized to be members of a large collection of genetic elements collectively termed the”mobilome” that constitute important but variable parts of most examined genomes [1]. In this view, the genomes consist of a fixed “core genome” and a collection of variable genetic elements that include plasmids, viruses, transposons, insertion sequences, integrative conjugative elements (ICE), as well as a large number of related sequences that are not always easily recognized. The mobilome, important for all organisms, has played an especially critical role in shaping the prokaryotic world. A testament to the significance of the mobilome is the conclusion that transposases, the genes that confer mobility to transposable elements, are the most ubiquitous genes in nature [2].

Insertion sequences (IS elements) are the smallest autonomous transposable genetic elements found in bacteria. They often consist of little more than one or two reading frames encoding a transposase, the enzyme required for transposition, as well as distinctive terminal sequences that set the IS element apart from the flanking host DNA [1, 3]. Most bacteria, including *Escherichia coli*, not only harbor more than one type of IS element, they often have multiple copies of many IS elements. The ISfinder database (http://www-is.biotoul.fr/) lists more than 4,000 different IS elements, and even this number represents only a fraction of the total number of IS elements found in publicly-available sequence databases.

Given their ubiquity and known roles in gene activation and inactivation, it is hardly surprising that a great many important roles have been ascribed to IS elements in shaping the bacterial genome [1]. For example, the presence of multiple copies of many IS elements furnishes regions of “portable homology” that are acted upon by host recombination functions leading to deletions and rearrangements in the bacterial chromosome as well as integration of exogenous DNA into the chromosome. IS element expansion can occur in nutrient-rich niches in which many genes become non-essential, and can therefore be inactivated, followed by genome contraction by deletions mediated by recombination at repetitive IS element copies. These events have been proposed to be important in the evolution of symbionts as well as intracellular pathogens.

While deduced expansion-contraction cycles offer dramatic examples of the role of IS elements in shaping the bacterial genome, there are many examples in which IS-mediated gene-activation and inactivation confer adaptive advantage to transient changes in the environment. This view is strongly supported by such well-known adaptive responses as phase and antigenic variation by invertible sequences (related to IS elements) in *Salmonella*, *E*. *coli* and other pathogenic bacteria (reviewed in [4]). More interesting still are examples where phase variation occurs by the precise insertion and excision of “normal” IS elements, as in a case involving the insertion of IS*492* into the *eps* locus (capsule synthesis) of the marine bacterium *Pseudomonas atlantica* [5], as well as a case for the insertion of IS*1301* into the *siaA* gene of the human pathogen *Neisseria meningitidis* [6]. In each of these cases, insertion occurs into a single hotspot, leading to inactivation of the operon. Subsequent precise excision restores the sequence and original level of operon expression.

In the examples cited above, the tacit assumption has been made that the underlying IS insertion and excision events are stochastic, with the environment playing no role in regulating these events, an assumption that to our knowledge has not been rigorously tested. Challenging the idea that IS hopping is always stochastic, is the emerging evidence that IS insertions can be selectively targeted to specific chromosomal loci by environmental stress conditions, and such targeting has the effect of relieving the stress. This concept, sometimes referred to as “directed” mutation, is controversial because it has been misinterpreted as invoking a Lamarckian process. However, as considered further elsewhere in this communication, the underlying processes do not violate fundamental genetic principles, but merely illuminate previously under-appreciated aspects of the evolved relationship between transposons and their host genomes. Table 1 is a non-exhaustive list of experimental systems that have been used to suggest that IS element insertion is elevated under stressful conditions. In this communication we focus on five systems in which IS*5* insertion leads to gene activation or inactivation.

**Table 1:** Bacterial experimental systems in which environmental conditions are known (or proposed) to influence the frequencies of IS insertions that activate or inactivate genes.

Organism	operon	Stress	IS element	Reference
*Escherichia coli*	*glpFK* activation	starvation in the presence of glycerol	IS*5*	[7–9]
*Escherichia coli*	*flhDC*	Nutrient depletion on motility agar	IS*5*, IS*1*, IS*3*	[10], [11]
*Escherichia coli*	*nfsB*	Furazolidone toxicity	IS*5*, IS*3*, IS*1*	[12]
*Escherichia coli*	*bglG*	starvation in the presence of arbutin or salicin	IS*5*	[13]
*Escherichia coli*	*fucAO-fucPIK*	Starvation in the presence of L-1,2-propanediol	IS*5*	[14, 15]
*Cupriavidus metallidurans*	*cnrYX*	Zinc toxicity	IS*Rme5*, IS*Rme3*, IS*Rme15*, IS*1090*, IS*1088*, IS*1086* and IS*1087B*	[16]
*Escherichia coli*	*cel*	starvation in the presence of arbutin or salicin	IS*1*, IS*2*, IS*5*	[17]
*Escherichia coli*	*asc*	starvation in the presence of arbutin or salicin	IS*186*	[18]
*Escherichia coli*	*ebgR*	Starvation in the presence of lactulose	IS30, IS1	[19]

Transposable genetic elements were long assumed to have little preference for a specific DNA target sequence, but more recent evidence has suggested that subtle structural features are important in target-site acquisition [3, 20, 21]. For example, in the *E*. *coli* genome, a preference is seen for intergenic regions. This situation could have arisen because selection preserves only those insertions that are either neutral or beneficial. It is additionally possible that transposition target selection favors specific structural features [1, 20]. For example, AT-rich sequences were proposed to be preferential targets for transposition. Another example is provided by transposons that appear to target replication forks, showing a preference for inserting into the lagging strand template [22]. Insertion mechanisms of many transposable elements involve introducing staggered cuts at short sequences (2–14 bp) leading to “target site duplication” (TSD), often considered to be the signature of transposition. Indeed, for a few transposons there is a high degree of target sequence specificity: for example, Tn7 has a single preferred site in the *E*. *coli* genome, although it can be forced to insert at certain other regions [23]. However, for a majority of transposons, the short sequences are neither highly conserved nor rare, and they therefore are unlikely to account for non-random target selection. Thus, DNA structural features outside of the potential target sites may play important roles [1].

A number of non-canonical DNA structures such as A-DNA, Z-DNA, H-DNA, G-quartets, SIDD (superhelical stress-induced DNA destabilization) regions and other “non-B DNA structures” have been identified in bacterial as well as eukaryotic genomes [24, 25]. Here we describe an association between IS*5* element insertion and SIDD sequences in experimental systems in which environmental stress leads to increased transposition. With specific focus on IS*5*, we propose and test a speculative model on the roles of DNA structure and DNA binding proteins on transposition target selection, and how target selection could be affected by environmental stress.

## Materials and methods

### SIDD mapping of DNA sequences

All analyses were carried out on *Escherichia coli* K12 (MG1655 or BW25113) DNA sequences from GenBank (Table 2). Each sequence was between 4 to 5 kb long, and contained the gene in question at the center along with a 2 kb sequence upstream of the ATG start codon, and the 2 kb sequence downstream of the stop codon. SIDD regions in each sequence were mapped using the SIST program with default parameter settings (superhelical density σ = -0.06, temperature, 37 ^o^C, ionic strength = 0.01 *M*; type of molecule = linear) [26–30].

**Table 2:** Sequence co-ordinates of DNA sequences used for SIDD mapping.

*E*. *coli* Strain	Operon	Starting base#	Ending base#	Fragment Size (bp)
MG1655	*glpFK*	4,115,344	4,120,089	4,846
	*bglGFB*	3,903,830	3,908,566	4,837
	*fucAO*	2931139	2,935,688	4,648
	*nfsB*	602869	607,424	4,654
BW25113	*flhDC*	1,970,104	1,974,454	4, 351

### Construction of *nfsB* alleles with altered SIDD profiles

We used a recombineering protocol based on GalK positive-selection and counter-selection [31] to modify the *nfsB* gene *in situ*. To construct the DNA duplex-stabilizing *PROM-3* mutation, three bases, AA (+6 and +7 from *nfsB* transcription start) and T (+14 from *nfsB* transcription start), were changed to CC and G, respectively (the larger sequence context for these changes is shown in Results and Discussion). To accomplish this, first the *galK* gene together with its constitutive em7 promoter (em7-*galK*) was amplified from the pGalK plasmid [31] using primers GalK-PROM3-P1 and GalK-PROM3-P2, each of which is composed of a 20 bp region at its 3’ end that is complementary to the em7-*galK* sequence, and a 50 bp region at its 5’ end that is homologous to the *nfsB* gene (see S2 Table). The PCR products were gel purified, treated with *Dpn*I, and then electroporated into BW25113 Δ*galK* cells (Gal^**-**^) expressing Lambda-Red proteins encoded by plasmid pKD46 [32]. The cells were plated on minimal M9 agar plates with galactose (0.5%, w/v) as the sole carbon source. After 3 days of incubation at 30 ^o^C, several colonies (Gal^**+**^) were purified, and subsequently verified for the replacement of the 9-bp region (AAATTACTT) located between +6 and +14 by PCR followed by DNA sequencing. A resulting strain was named BW_K.

A 100-bp synthetic DNA oligonucleotide with the sequence (ctcgcttaccatttctcg ttgaaccttgtaatctgctggcacgcaa**CC**ttact**G**tcacatggagtctttatggatatcatttctgtcgccttaaagcgtc) that contained substitutions of three nucleotides (boldfaced and uppercase letters) was PCR amplified. The DNA products were purified and subsequently electroporated into BW_K cells expressing the Lambda-Red proteins. After incubation at 30 ^o^C for 1 h, the cells were pelleted, washed once by resuspension in 1x M9 salts, and subsequently spread on M9 agar supplemented with 0.2% glycerol and 0.2% 2-deoxygalactose (a galactose analog that is toxic when it is phosphorylated by GalK). After 3 to 4 days of incubation at 30 ^o^C, about 10 colonies (Gal^-^) were purified using the same agar plates and then verified for the replacement of em7-*galK* with the 100-bp DNA fragment (containing the desired 3 nucleotide substitutions) using PCR and subsequent DNA sequencing.

To construct the DNA duplex-destabilizing *ORF-3* mutation, three bases, C (+38 from *nfsB* transcription start), G (+56 from *nfsB* transcription start) and G (+71 from *nfsB* transcription start) were each changed to the nucleotide “A” using the similar methods as above. The region containing the sequences “CATTTCTGTCGCCTTAAAGCGTCATTC CACTAAG” (bases +38 to +71 from *nfsB* transcription start) in strain BW25113 Δ*galK* was first replaced by em7-*galK*. A 100-bp DNA fragment (caaaattactttcacatggagtctttatggatat**A**atttctgtcgccttaaa**A**cgtcattccactaa**A**g catttgatgccagcaaaaaacttaccccg) that contained the desired 3 bases (boldface and uppercase letters) was then substituted for this em7-*galK* region. The larger sequence context for these changes is shown in Results and Discussion.

### Isolation and sequence analyses of furazolidone-resistance (FZD^r^) mutations in the *nfsB* gene

Three strains, BW25113 (Δ*nfsA*, *nfsB*^*+*^ carrying a wild-type SIDD region of the *nfsB* gene), *PROM-3* (Δ*nfsA nfsB*^*+*^ carrying mutations that increase duplex stability in the SIDD region of the *nfsB* gene) and *ORF-3* (Δ*nfsA nfsB*^*+*^ carrying mutations that further decrease duplex stability in the SIDD region of the *nfsB* gene), were used for isolation of FZD^r^ (Δ*nfsA nfsB*) mutants. Overnight cultures grown at 30 ^o^C in LB medium were diluted to an OD_600_ of 2. The diluted cultures (100 μl) were spread on LB agar plates containing FZD (6 or 8 μg/ml). The plates were incubated at 30 ^o^C for 36 to 40 h. The FZD^r^ colonies were counted. At least 100 colonies for each tested strain were picked onto new LB + FZD plates that were then incubated at 30 ^o^C overnight. To examine which FZD^r^ mutants are IS insertional mutants, the region containing *nfsB* and its promoter from over 100 FZD^r^ mutants derived from each strain was amplified by PCR using primers NfsB-ver-F and NfsB-ver-R (S2 Table). The PCR products were subjected to agarose gel electrophoresis, and any mutant with a ~2kb DNA band (as compared to a 1 kb band for wild type) was considered to be an IS insertion mutant. To identify which IS insertional mutants bore IS*5* elements, two rounds of PCR were performed. The first round of PCR used primers NfsB-ver-R and IS*5*-ver-F (specific to IS5 and oriented in the same direction as the transposase gene *ins5A*). Any IS*5* insertional mutant in the direct orientation would result in a ~ 1-kb dominant band. The second round PCR used primers NfsB-ver-F and IS*5*-ver-F. Any IS*5* insertional mutant in the reverse orientation would result in a dominant band (about 1 kb in size dependent on the IS5 locations). IS*5* insertional frequency was calculated by dividing the sum of IS*5* mutants from these two rounds of PCR by the number of the total FZD^r^ mutants.

## Results and discussion

### Stress-induced DNA duplex destabilization (SIDD) regions

In most autonomous organisms studied to date, DNA is maintained in a negatively supercoiled, or under-wound, state through the combined actions of topoisomerases, DNA-binding proteins, and enzymes responsible for transcription and DNA replication. In *E*. *coli*, topoisomerase II (also known as DNA gyrase), and to a lesser extent, topoisomerase IV, are the principal enzymes that cut, unwind and re-ligate DNA strands, thereby reducing the linking number. The stress of under-winding is relieved by supercoiling as well as other localized changes in DNA structure. A second set of topoisomerases, exemplified in *E*. *coli* by topoisomerase I (ω-protein) counteract excessive under-winding by re-winding DNA strands so that DNA is maintained in a slightly under-wound state. DNA transactions such as transcription and replication introduce positive supercoils ahead of the transcription bubble or replication fork. Transcription in particular is believed to be a major driver of the superhelical state of DNA because it produces positive supercoils ahead of the transcription bubble, and negative supercoils in its wake [33]. In addition, DNA-binding proteins such as histones in eukaryotes and histone-like nucleoid proteins in bacteria sequester and maintain superhelical domains [33]. The negative superhelical status of intracellular DNA is the sum of the contributions of topoisomerases (the unconstrained portion), and those of DNA-binding proteins such as histones or nucleoid proteins (the constrained portion). In actively dividing *E*. *coli* cells, negative supercoiling is maintained in the range of around σ = -0.06 (σ is the specific linking difference, or superhelical density; [34, 35]), with σ ranging under different conditions, from -0.03 to -0.09. Within this range, the supercoiling status is regulated in part by the intracellular energy charge of the adenylate pool, approximated as the ATP/ADP ratio [36].

The energy stored in negative supercoils is not only essential for DNA transactions such as transcription and replication, but also drives localized sequence-dependent structural transitions with important biological consequences. Such superhelical stress-induced structural transitions (SIST) include SIDD regions, Z-DNA, cruciform extrusions, H-DNA, and G-quadruplexes. Benham and co-workers have developed statistical-mechanical computational procedures capable of accurate predictions of SIDD regions grounded on experimentally determined DNase-sensitivity data [26–29]. They have applied these computational methods to map SIDDs as well as other DNA structural transitions such as H-DNA, Z-DNA and cruciform structures in the entire *E*. *coli* genome [25]. They have further shown that SIDD sequences are associated with both transcriptional regulatory sequences [26, 37], and eukaryotic replication origins [38]. Their analysis of *E*. *coli* K12 genomic DNA showed that about 1% of the genomic DNA is strongly destabilized, 12% moderately destabilized, and that >75% of genomic DNA is stable under physiological levels of superhelical stress [37].

In this communication, we have analyzed all five known experimental systems in which IS*5* insertion events occurred under conditions of starvation or nutrient depletion (*glpF*, *bglG*, *fucA* and *flhD*) or under antibiotic stress (*nfsB*), and in which sites of IS*5* insertion have been determined at the sequence level. Here we summarize salient features of each system, along with an analysis of the DNA structural context of IS*5* insertions.

### IS*5*-mediated activation of the glycerol utilization operon *glpFK* under starvation stress

The best-characterized experimental system in which environmental conditions appear to stimulate IS*5* hopping is the *E*. *coli glpFK/*Crp experimental system [7, 8]. In wild type *E*. *coli* cells, glycerol utilization requires expression of the genes that are normally poorly expressed in the presence of glucose. The first operon in the glycerol utilization pathway, *glpFK*, codes for the glycerol uptake facilitator, GlpF, and the kinase, GlpK, that phosphorylates glycerol to glycerol-3-phosphate (G3P). G3P has two functions: it is the inducer of the five-operon *glp* regulon, and it feeds into glycolysis after conversion to dihydroxyacetone phosphate, catalyzed by G3P dehydrogenase (GlpD). The *glpFK* operon is repressed by the binding of the GlpR repressor to four binding sites (operators; *O*_1_ –*O*_4_) in the promoter region, and is activated by the binding of the cAMP-Crp complex to two binding sites that overlap two of the GlpR binding sites, *O*_2_ and *O*_3_ [7]. G3P binds to and releases GlpR from the DNA, and concomitant binding of the cAMP-Crp complex to the two binding sites (*O*_CrpI_ and *O*_CrpII_) in the promoter activates the transcription of the *glpFK* operon. Thus, cells lacking either the *crp* (Crp), or the *cyaA* (adenylate cyclase; biosynthesis of cAMP) gene are Glp^-^ because the *glpFK* operon is essentially silent, and do not form colonies on glycerol minimal agar plates because they cannot utilize glycerol as a carbon source.

Prolonged incubation of Glp^-^ cells on glycerol minimal agar plates, however, led to the continuous generation of Glp^+^ colonies starting from day 3 and extending over a period of 9 days. In every case, IS*5* is found to be inserted upstream of the *glpFK* promoter at the same specific location and in the same orientation. Subsequent work established several key features of this *glpFK*-activating insertional mutation. (1) The mutation leads to high-level activation of the native *glpFK* promoter [7, 8]. (2) Presence of glycerol is necessary to stimulate IS*5* insertion in *glpR*^+^ cells, but not in Δ*glpR* cells. Moreover, IS*5* insertion was significantly decreased upon over-expression of GlpR, indicating that GlpR binding hinders IS*5* insertion. (3) GlpR inhibition of IS*5* insertion is independent of the role of GlpR in repressing *glpFK* operon transcriptional expression [7]. (4) Increased IS*5* insertion due to the presence of glycerol is specific to the *glpFK* promoter, and is not observed at other tested loci where insertion events can be monitored [7]. Furthermore, the mutagenic process could be demonstrated independently of the selection procedure [7, 39]. (5) IS*5* insertion into the *glpFK* promoter region is blocked by the binding of the cAMP-Crp complex to its two operators (*O*_CrpI_ and *O*_CrpII_) [39].

Any hypothesis that seeks to implicate an environmental condition in controlling beneficial IS insertion at a specific locus requires a plausible chain of molecular events linking the environmental condition to the transposition event. Despite the wealth of information available about IS*5*-activation of the *glpFK* operon, as summarized above, the mechanisms that promote IS*5* insertion at this specific locus have not been explored.

**Fig 1:**
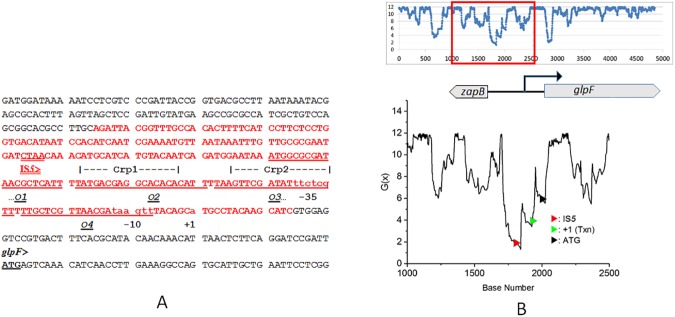
**Panel A**. The *zapB*-*glpF* intergenic region showing the position of IS5 insertion and its orientation. The transcriptional start site is indicated as +1. The SIDD region (DNA sequences at which G(x) values fall below 6 kcal/mol) is shown in red. The four operator sequences labeled *O*_1_ through *O*_4_ are underlined, and the two Crp-cAMP binding sites that overlap *O*_2_ and *O*_3_ are indicated by dashed lines above the sequence. The CTAA sequence at which IS*5* insertion occurs is underlined (> indicates IS*5* orientation). **Panel B**. Plot of G(x) values (see *Materials and Methods* and Table 2) for a 4.8 kb DNA sequence from *E*. *coli* K12 MG1655. The entire plot is shown at the top, and for clarity, a segment of the plot (bp 1000 to 2500) is expanded at the bottom. The locations of the IS*5* insertion (red arrow), the start sites for transcription (green arrow) and translation (black arrow) are shown on the energy plot.

Fig 1 shows an analysis of a 4.8 kb segment of the *E*. *coli* MG1655 genome encompassing the *glpFK* locus, obtained using the SIST codes generously made available by Prof. C. J. Benham. The destabilization energy G(x) is the incremental energy (in kcal/mol) required to ascertain that base pair x will be unpaired under physiological conditions of superhelical density (σ = -0.06), temperature (37 ^o^C), and ionic strength (0.01 *M*). Stable duplex DNA regions have high G(x) values (about 12 kcal/mol in Fig 1), whereas regions susceptible to strand separation have lower G(x) values. In Fig 1, panel B, a G(x) plot for the entire 4.8 kb fragment, is shown at the top, with a segment expanded underneath to show greater detail. In Fig 1, panel A, the red text identifies base pairs where G(x) values fall below 6 kcal/mol. Fig 1 shows that the activating IS*5* insertion in the *glpF* upstream region targets a CTAA tetranucleotide embedded within a sequence of very low G(x) values (dipping below 2) within an extended SIDD region.

The occurrence of the *glpFK*-activating IS*5* insertion within a SIDD sequence offers an opportunity to address two questions: (1) How can one possibly link an environmental stress condition (carbon starvation in the presence of glycerol this case) to IS*5* hopping into a specific locus, and (2) What exactly is the role of low G(x) values on target selection for IS*5* insertion?

We propose that the CTAA sequence embedded within the SIDD region constitutes an IS*5* insertional hotspot that is, however, masked and remains inaccessible in wild type cells under normal growth conditions. During growth in rich or defined glucose medium, the *glpFK* operon is repressed by the binding of the GlpR repressor protein to the DNA sequences upstream of the *glpF* promoter, and this binding serves to mask the IS*5* site in the sense to be discussed below. Under conditions where glycerol is the sole carbon source, in normal wild type (*crp*^*+*^) cells, cAMP synthesis leads to an accumulation of the cAMP-Crp complex which then binds to the same upstream region as the GlpR protein, and activates the *glpFK* operon. The initial burst of GlpF and GlpK synthesis leads to facilitated glycerol uptake and phosphorylation to produce G3P, which then physically binds to and inactivates GlpR, thereby allowing the expression of the *glpFK* operon. In these cells, even though GlpR has been inactivated, occupation of the same DNA region by the cAMP-Crp complex continues to mask the IS*5* site. When either the *crp* gene, or the *cyaA* (cAMP biosynthesis) gene is deleted, there is no cAMP-Crp complex to mask the IS*5* insertion site, but GlpR is still present and continues to mask the insertion site. Support for this idea is provided by the finding that in Δ*glpR* cells, IS*5* insertion rates are elevated some 100-fold by the loss of the cAMP-CRP complex in cells growing in rich liquid medium (LB), and the presence or absence of glycerol in the medium has no effect; conversely, over-expression of GlpR essentially eliminates IS*5* insertions [7, 39].

**Fig 2:**
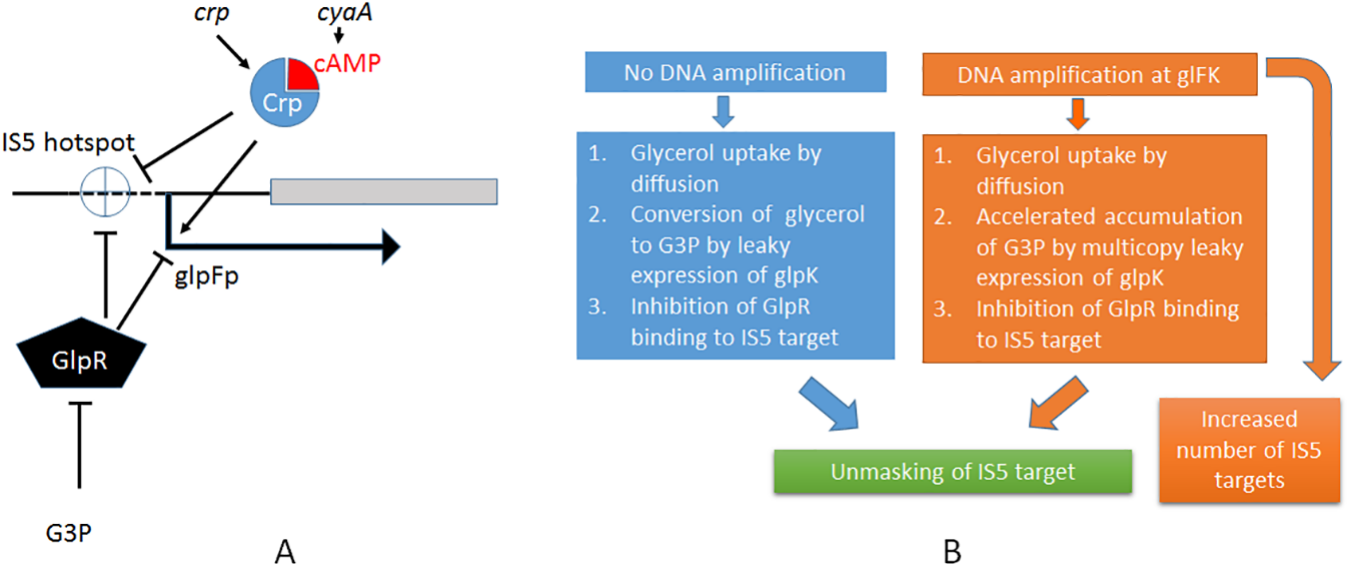
**Panel A**. Role of transcription factors Crp-cAMP and GlpR on *glpFK* expression, and on IS*5* insertion. Crp, when complexed with cyclic AMP (cAMP), activates transcription (→) but inhibits IS*5* insertion (--|). GlpR in the free form inhibits both transcription, and IS*5* insertion(--|), but when present in sufficient quantities, glycerol 3-phosphate (G3P) binds to GlpR, inducing a conformational change that causes the protein to dissociate from the DNA, relieving the inhibition of transcription, and allowing IS5 insertion [7, 39]. **Panel B**. Models for unmasking of the IS*5* insertion hotspot with and without regional DNA amplification. When Δ*crp* or Δ*cyaA* cells are subjected to prolonged incubation on minimal agar with glycerol as the sole carbon source, some glycerol diffuses into cells, and is converted to G3P at low efficiency (see text). Accumulation of G3P over time leads to dissociation of GlpR from the *glpFK* promoter region, unmasking the IS*5* insertion hotspot. In an alternative scenario, IS*5* insertion occurs in a subset of cells in which the *glpFK* region is amplified, leading to accelerated release of GlpR due to multicopy titration of GlpR. Note that the gene encoding GlpR is located 12 minutes away, and is unlikely to be co-amplified with *glpFK* in this scenario. Furthermore, to the extent that G3P formation depends on leaky expression of *glpK*, amplification will also lead to a more rapid accumulation of G3P. Finally, multiple copies of the IS*5* target site resulting from *glpFK* amplification increase the likelihood of capturing an insertion event in these cells.

We propose that prolonged incubation of Δ*crp* cells on glycerol minimal agar leads to a gradual release of the GlpR repressor from its operators, and the consequent unmasking of the IS*5* site (Fig 2A). Although GlpF facilitates glycerol uptake, it is known that glycerol can also passively diffuse into the cell from the medium [40]. We propose that this small pool of glycerol gets phosphorylated to G3P either due to low-level (leaky) expression of the *glpK* gene or due to the activity of an unknown sugar kinase capable of acting on glycerol with low efficiency. When G3P levels build to a threshold concentration, GlpR is inactivated and the IS*5* site is unmasked (Fig 2A).

There is at least one other mechanism that can achieve a similar effect in a subset of cells. Roth and co-workers have argued that when *E*. *coli* FC40 (F’ *lac*^*-*^) cells are subjected to prolonged incubation on lactose minimal agar, in discrete subpopulations of non-dividing cells, the F’ plasmid copy numbers increase, and the higher gene copy numbers provide greater opportunities to acquire a random mutation [41]. If a *lac*^+^ reversion does arise in some cells with multiple *lac*^-^ gene copies, then those cells will divide using lactose, and eventually form stable *lac*^+^ colonies. The idea of selective gene amplification is directly extensible to chromosomal loci because discrete sub-populations of starving cells are known to have amplified different chromosomal segments [41, 42]. Extending this idea to the *glpFK*/Crp experimental system, one proposes that in a subset of cells, regional amplification of the *glpFK* locus creates multiple copies of the gene without co-amplifying the *glpR* gene which is encoded in a distant chromosomal locus 12 minutes away, and therefore, leads to a simple titration of the cellular GlpR concentration. Lack of sufficient GlpR in turn promotes IS*5* insertion both by unmasking the target site, as well as by providing multiple copies of the unmasked target site in the same cells (Fig 2B). The most interesting aspect of these speculative mechanisms is that they provide a conceptual framework for mutation directed by a specific stress environment. While these mechanisms remain to be experimentally verified, it is evident that each mechanism yields testable predictions.

We next consider what constitutes an IS*5* hotspot, and what roles SIDD sequences might play in creating a hotspot. IS*5* insertion leads to the duplication of a short tetranucleotide sequence with the consensus sequence Py-T-A-Pu, although the most common sequence is CTAG, which is an under-represented tetranucleotide in bacterial genomes [43]. However, as shown in Table 3, the sequence requirement is not strict, and one or the other known target tetranucleotide sequence is observed approximately once every 50 bp. Thus a target sequence plays a role, but additional DNA (structural) features are apparently required for insertion [3, 20, 21].

**Table 3:** Known tetranucleotide target sequences for *IS5* (from ISfinder).

Tetranucleotide	Number of occurrences
CTAG	15
CTAA	3
TTAG	3
CAAG	1
CTTA	1
CTTT	1
Total	24

Source: https://www-is.biotoul.fr/scripts/ficheIS.php?name=IS5

Although the IS*5* transposition mechanism has not been directly investigated, IS*5* encodes a “DDE type” transposase, which is by far the most common as well as the best-studied class of bacterial transposases. Target acquisition by Mu transposase, a well-studied example of a DDE transposase, requires significant 140^o^ bending of the target sequence [44]. Even though there are substantial differences in the detailed mechanisms of different DDE type transposases, DNA bending (deformation) appears to be a general feature of this class of transposases. The significance of SIDD sequences could be that they are easily deformed when their duplex status is not stabilized by DNA binding proteins such as GlpR or cAMP-Crp. Thus, an easy-to-deform sequence context (such as a SIDD sequence) surrounding a Py-T-A-Pu target tetranucleotide might constitute an IS*5* hotspot.

SIDD sequences might constitute an attractive IS*5* target for other reasons as well. As noted previously, although SIDD sequences are AT-rich, AT-richness, as well as the actual sequence and the sequence context determine the depth and width of a SIDD destabilization “well” [27, 28, 37]. It has been shown that the *E*. *coli* nucleoid protein H-NS (encoded by *hns*) binds to AT-rich sequences and H-NS binding often leads to gene-silencing [45, 46]. Interestingly, H-NS was found to be required for efficient transposition of several transposons, including IS903, Tn10 and Tn552 as transposition rates are significantly reduced in Δ*hns* cells [47]. Thus, H-NS bound to a SIDD sequence might be an important feature in generating an IS*5* hotspot. We speculate that the inhibition of IS*5* transposition by GlpR and cAMP-Crp may be mediated partly by exclusion of H-NS, and partly by their ability to stabilize the DNA duplex structure. Thus, a favored IS*5* target sequence might be a Py-T-A-Pu tetranucleotide embedded in a SIDD sequence because such sequences can (1) be easy-to-deform, and/or (2) have the potential to bind H-NS.

Other possible roles for SIDD sequences cannot be eliminated. For example, some transposable elements insert into transient single-stranded regions created during replication [48]. The lower energetic cost of melting duplex DNA within a SIDD sequence may thus create similar transiently single-stranded targets. It is also possible that DNA melting at SIDD sequences enables binding by HU, another nucleoid protein, which in turn may play a role in transposition of some elements such as bacteriophage Mu [44].

### Activation of *bglGFB*, the cryptic aromatic β-glucoside utilization operon

The *E*. *coli bgl* system offers a well-characterized early example of gene activation by transposon insertion [13, 49–51], but it differs from the *glpFK* system in two key characteristics: whereas IS-hopping in the *glpFK*/Crp system occurs at a single locus, and is promoted in non-growing or slowly-growing cells, IS insertion in the *bgl* system occurs at several loci, and occurs both in dividing cells and in non-dividing cells, and therefore offers a somewhat different paradigm. The *bglGFB* operon is cryptic, but when activated by IS insertion, its expression enables cells to utilize aromatic β-glucosides such as arbutin and salicin. The operon is regulated by a transcription-attenuation mechanism such that *bglG*, the first gene in the operon, must be expressed in order to overcome the transcriptional attenuation. In wild type cells, the *bglG* promoter is flanked by inhibitory DNA sequences that prevent expression of *bglG*. Two classes of mutations can activate the *bgl* operon: the first and major class involves insertion of an IS element in the promoter-flanking inhibitory sequences, and the second and minor class involves mutational inactivation of *hns*, the gene encoding the histone-like nucleoid protein H-NS [13, 51, 52]. In wild type cells, H-NS is believed to silence the *bglG* promoter by binding to the promoter sequences, and thus, IS insertions and *hns* mutations both appear to act by relieving H-NS repression. Reynolds *et al*. showed that the *bgl* operon is activated by transposon-hopping in the *bglG* promoter region at exceptionally high frequencies (10^−5^), almost always by the insertion of IS*5* or IS*1*, but less frequently, also by IS*2* and IS*3* [52], suggesting that this DNA region constitutes a transposition hotspot. In a subsequent study, Hall showed that when wild type (Bgl^-^) cells are subjected to prolonged incubation on minimal media with arbutin as the sole carbon source, Bgl^+^ colonies arise from non-dividing cells, apparently at high frequencies. A major fraction of these so-called adaptive mutations were due to insertion of IS*1* or IS*5* within the same 19 bp hotspot found previously in dividing cells [49].

**Fig 3:**
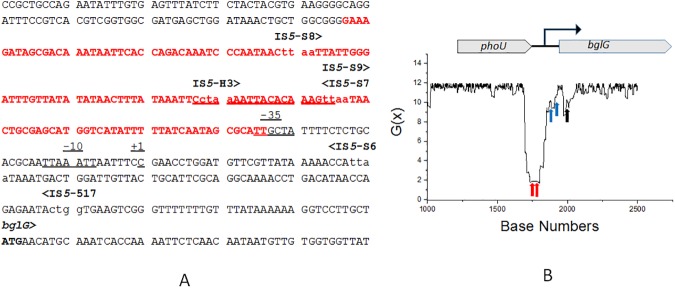
**Panel A.** The DNA sequence of the *phoU*-*bglG* intergenic region showing IS*5* insertion sites, and the DNA sequence at which G(x) values fall below 6 (the SIDD zone). The positions of the insertion sites for the IS*5* element are shown in lower case letters (ttaa, ctaa and ctgg), and orientations are shown by > or <. Note that IS*5*-S6 and IS*5*-517 are outside of the indicated SIDD zone (*see text*). The transcriptional start site is indicated as +1. **Panel B**. Plot of G(x) values (see *Materials and Methods* and Table 2) for a 4.6 kb DNA sequence from *E*. *coli* K12 MG1655. For clarity, only a segment of the plot (bp 1000 to 3000) is shown. The location of two IS*5* insertion sites representing two of the most frequent events are indicated by red arrows, and the much less-frequent outlier insertion sites (IS*5*-S6 and IS*5*-517) are shown by blue arrows (*see text*). The translational start site for *bglG* is indicated by a black arrow.

Fig 3 shows an analysis of a 4.8 kb segment of the *E*. *coli* MG1655 genome encompassing the *bglG* locus using the SIST codes. In Fig 3, panel A, the red text identifies base pairs where G(x) values fall below 6, and panel B shows the G(x) energy plot of the region. It is immediately apparent that most IS insertions in growing cells [13] as well as starved cells [49] fall within the SIDD region, as also shown graphically using red arrows in panel B. In addition to a large number of insertions that occurred in the -70 to -150 region (relative to the transcriptional start site in Fig 3), Schnetz and Rak [13] identified two rare IS*5* insertions isolated from growing cells that fell downstream of the *bglG* promoter (blue arrows in panel B). Schnetz and Rak reported that these downstream insertions occurred at a frequency of <0.01% of the Bgl^+^ mutants. We interpret this finding to mean that even though insertions outside of the SIDD region could confer the Bgl^+^ phenotype, most insertions however were targeted to SIDD regions, strengthening the correlation that insertion events were indeed favored in the SIDD region.

The exceptionally high frequencies of IS*5*-hopping at the *bgl* locus suggest that H-NS binding to promoter-flanking sequences creates constitutive IS*5* hotspots at several Py-T-A-Pu sequences embedded in the SIDD region. It implies that no duplex-stabilizing proteins equivalent to GlpR bind to these constitutive hotspots in laboratory growth conditions. The *bglG* upstream region does have binding sites for Fis, another nucleoid protein, and binding sites for the transcription-activating proteins such as Crp, RcsB/BglJ and LeuO [53]. Whether these proteins impact IS-hopping remains to be investigated.

### Activation of swarming motility by IS element insertion into the *flhD* promoter region

In *E*. *coli*, swarming motility is believed to confer survival advantages in toxic or nutritionally poor environments, and requires the expression of flagella. The flagellar synthesis regulon, a very large and complex system consisting of some 50 genes [54], is regulated by the two master regulators, FlhD and FlhC expressed from the *flhDC* operon. In many strains of *E*. *coli*, such as BW25113, the *flhDC* operon, whose expression is under the control of an exceptionally complex regulatory region, remains largely cryptic such that flagellar expression is low, and the cells do not swarm when grown in either liquid media where swarming confers no benefit, or on solid agar media where swarming is disallowed. Two recent studies demonstrated however that when plated on semi-solid agar media (“motility agar”) where swarming is allowed, mutants capable of increased swarming ability appear [10, 11], and their emergence accelerates as the cells approach stationary phase [11]. The activating mutations are due to the insertion of IS elements in the *flhDC* upstream regulatory region. These findings have been interpreted to mean that specific environmental conditions promote mechanisms that lead to beneficial mutations in the *flhDC* operon. Because such mutations do not occur (or occur at very low levels) when not beneficial, as in the case of growth in liquid medium where swarming is unnecessary, or growth on solid agar where swarming is disallowed, mutations that arise only when useful are proposed to be examples of “quasi-Lamarckian” [10] or “directed” [11] mutations.

**Fig 4:**
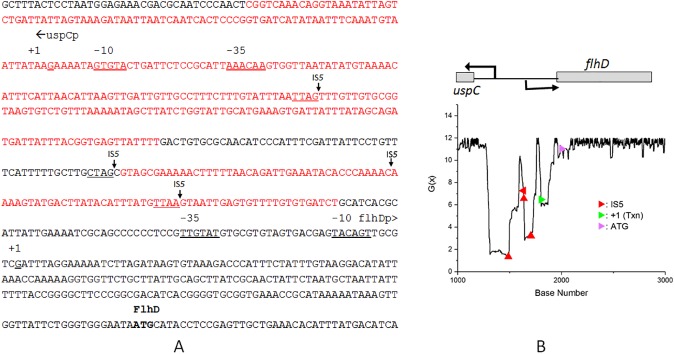
**Panel A.** The *uspC*- *flhDC* intergenic region in *E*. *coli* K12 BW25113 showing positions of IS*5* insertion sites and the SIDD sequences (red) G(x) values fall below 6. The positions of the insertion sites for IS*5* elements are shown (underlined TTAG, CTAG and TTAA). The transcriptional start sites for the two divergently transcribed genes are indicated as +1, and the *flhD* translational start site ATG is indicated. **Panel B**. Plot of G(x) values (see *Materials and Methods* and Table 2) for a 4.3 kb DNA sequence from *E*. *coli* K12 BW25113. For clarity, only a segment of the plot (bp 1000 to 3000) is shown. The locations of the IS5 insertion (red arrowheads), the start sites for transcription (green arrowhead) and translation (pink arrowhead) for the *flhD* gene are shown on the energy plot.

Analysis of a 4.4 kb sequence embedding the divergently transcribed *flhDC*-*uspC* intergenic region in *E*. *coli* BW25113 showed an extensively segmented SIDD region upstream of the *flhD* start codon (Fig 4, panel A). IS*5* insertion points occurred within the segmented SIDD sequence with G(x) values ranging from ~1 to ~7 as compared to a baseline value of 12 for the region shown. A number of negative regulatory proteins (RcsAB, YjjQ, OmpR, Fur, IHF, LrhA, MatA, H-NS), positive regulatory proteins (Crp) bind to this region [55–59]. Of note is the RcsAB protein, which, as a part of the RcsC phosphorelay system that responds to a variety of environmental inputs including surface-sensing [60], could influence DNA structural features relevant to transposon target acquisition. Regardless of whether there is a specific role for the RcsC phosphorelay system, the *flhDC* system offers an opportunity to examine if mechanosensing can influence transposition by influencing the structural states of DNA.

### Reversible activation of a cryptic anabolic function by IS*5* insertion

**Fig 5:**
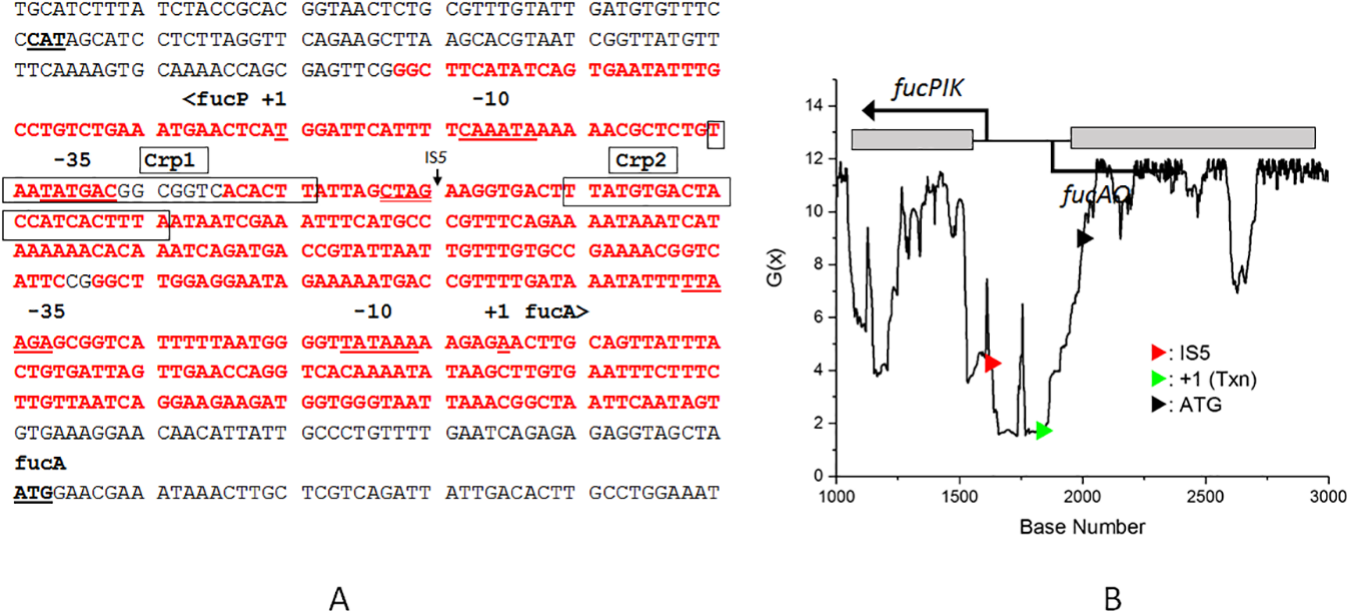
**Panel A.** DNA sequence encompassing the divergently transcribed *fucAO*-*fucPIK* intergenic region showing the single IS*5* insertion site and the SIDD zone of the same region (red) where G(x) values fall below 6. The single IS*5* insertion site CTAG (double-underline) is shown. The transcriptional start sites are indicated as +1, and the translational start site for *fucA* is indicted. **Panel B**. Plot of G(x) values (see *Materials and Methods* and Table 2) for a 4.6 kb DNA sequence from *E*. *coli* K12 MG1655. For clarity, only a segment of the plot (bp 1000 to 3000) is shown. The location of the IS5 insertion (red arrowhead), and the transcriptional (green arrowhead) and translational (black arrowhead) start sites for the *fucA* gene are shown on the plot.

Metabolism of fucose by wild type *E*. *coli* cells leads to production of the waste product, 1,2-propanediol (PPD), which is excreted into the medium. PPD cannot be used as a carbon source by wild type cells, but acquisition of an IS element in the intergenic region between the divergently transcribed *fucAO* and *fucPIK* operons confers the ability to use PPD as a sole carbon source [14]. However, this benefit comes at the cost of simultaneously losing the ability to utilize fucose. In wild type *E*. *coli* cells, utilization of fucose requires the sequential actions of a permease (*fucP*), an isomerase (*fucI*), a kinase (*fucK*) to yield the sugar phosphate, L-fuculose-1-phosphate. An aldolase (*fucA*) then converts this sugar phosphate to dihydroxyacetone phosphate plus L-lactaldehyde [14]. Under non-respiratory conditions, L-1,2-propanediol oxidoreductase (FucO), an iron-dependent group III dehydrogenase, converts L-lactaldehyde to PPD which is excreted. The inducer for the *fucAO*-*fucPIK* system is fuculose-1-phosphate. The lactaldehyde ⇔ PPD conversion by the *fucO* oxidoreductase is reversible, but in wild type cells, PPD cannot be utilized as a sole carbon source because the *fucO* oxidoreductase itself is not induced, and therefore unavailable. Prolonged incubation of wild type cells on PPD minimal agar, however, yields PPD^+^ mutants that can grow on PPD because the *fucAO* operon is constitutively expressed [14, 15]. All PPD^+^ mutants acquire an IS*5* element at a specific locus in the *fucAO*-*fucPIK* intergenic region (Fig 5, panel A). The insertion occurs at a CTAG sequence within the broad SIDD region which encompasses complex, but poorly-described, regulatory sequences for the divergent *fu*c operons. The acquisition of the IS element, while rendering the *fucAO* operon constitutive, also renders the divergently expressed *fucPIK* operon non-inducible, thus effectively rendering the cells PPD^+^, but fucose-negative (Fuc^-^). Zhang *et al*., showed that when PPD^+^/Fuc^-^ mutants were plated on fucose minimal agar, PPD^-^/Fuc^+^ back-mutants arose in which precise excision of the original IS*5* insertion had occurred [15]. We propose that transposition and precise excision of IS*5* might constitute a reversible adaptive system for competing with other enteric bacteria in the gut by acquiring the ability to utilize a carbon source that cannot be utilized by competing bacteria, as hypothesized for *Salmonella enterica* serovar Typhimurium [61]. The *fucAO*-*fucPIK* experimental system, by allowing selection for both insertion and precise excision of *IS5*, offers an opportunity for dissecting DNA structural features important for insertion as well as excision [15].

### IS-mediated gene-inactivation under antibiotic stress

Furazolidone (FZD) is a nitroheterocyclic aromatic compound that upon activation, damages DNA and kills bacteria [62]. Activation is believed to require the action of several nitroreductases, the most important of which are encoded by the genes *nfsA* and *nfsB*. The toxic products of FZD nitroreduction are believed to be hydroxylamine derivatives [63]. Resistance to FZD proceeds through two steps: in the first step, *nfsA* is inactivated by mutation leading to decreased sensitivity, and in the second step, *nfsB* is inactivated [62], leading to much greater resistance. In both genes, large fractions of the inactivating mutations are due to IS element insertions. In the *nfsB* gene, there is a strong hotspot for IS*5* insertion [64]. The *nfsB* system is especially interesting because inactivating mutations conferring an FZD-resistant phenotype could occur at a large number of known inactivating sites throughout the reading frame, but all IS*5* insertions occur at a single locus in the N-terminal portion of the reading frame. We therefore asked if this insertional hotspot correlates with a SIDD region.

**Fig 6:**
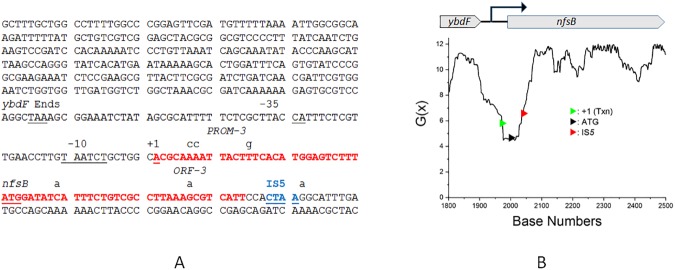
**Panel A.** The *ybdF*-*nfsB* intergenic region showing the position of the IS5 insertion site, and the SIDD sequences where G(x) values fall below 6 (red). The IS*5* insertional hotspot is shown in underlined blue letters within the N-terminal portion of the *nfsB* gene. The transcriptional start site (+1) and the translational start site for *nfsB* are indicated. The three transversions (two A-to-C, plus one T-to-G) constituting the Prom-3 mutant, and the three base changes (one C-to-A transversion, and two G-to-A transitions) constituting the ORF-3 mutant are indicated (*see text, and Fig 7*). **Panel B**. Plot of G(x) values (see *Materials and Methods* and Table 2) for a 4.6 kb DNA sequence from *E*. *coli* K12 MG1655. For clarity, only a segment of the plot (bp 1800 to 2500) is shown. The location of the IS5 insertion (red arrowhead) and those for the transcriptional (green arrowhead) and translational (black arrowhead) start sites are indicated on the plot.

Fig 6 shows an analysis of part of a 4.7 kb DNA segment of the *E*. *coli* MG1655 genome encompassing the *nfsB* gene. In Panel A, base-pairs with G(x) values of <6 are shown in red. The major IS*5* hotspot is a CTAA sequence (bases 38–41 in the N-terminal portion of the reading frame; blue lettering in Fig 6, panel A) that is in a region with G(x) values of 6.5, and located within the same SIDD “well”. Several other IS elements target the same SIDD region (not shown). Whiteway *et al*. [64] previously reported that *nfsB*-inactivating IS*1* and IS*2* insertions occurred at a number of sites throughout the *nfsB* reading frame. Multiple potential IS*5* target tetranucleotide sequences (Py-T-A-Pu) are observable throughout the *nfsB* reading frame, but, IS*5* insertion was not observed at these other sites. In our experiments, consistent with prior findings, the major insertion event was IS*5* insertion at the single location in the N-terminal region of the *nfsB* reading frame (blue lettering in Panel A, Fig 6).

**Fig 7:**
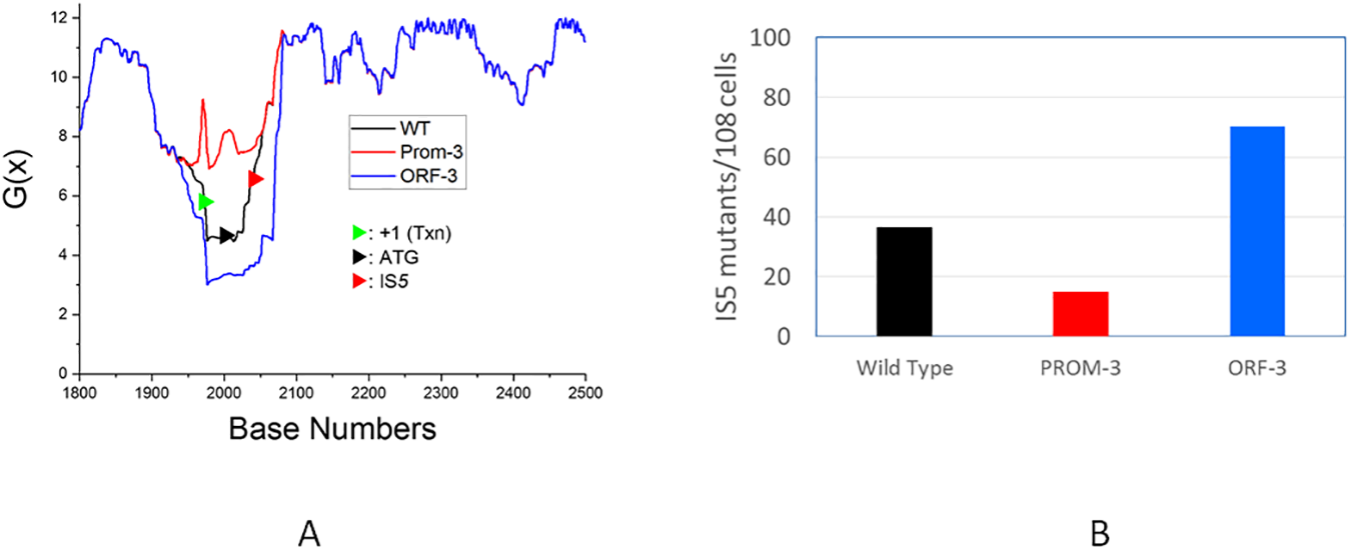
**Panel A.** Analysis of the effect of *in silico* mutations on the SIDD profile of the *ybdF*-*nfsB* intergenic region. As shown in Fig 6, the *PROM-3* mutant has three A:T to C:G transversions downstream of the transcription start site (+1 in Fig 6), whereas the *ORF-3* mutant has one G:C to T:A transversion, and two G:C to A:T transition mutations. SIDD plots for the wild-type (black line), *Prom-3* (red line) and *ORF-3* (blue line) sequences are shown. Relative to the wild type sequence, the G(x) values are increased in the *Prom-3* mutant (red), meaning that DNA duplex is more stable than wild type in this region. The G(x) values are decreased for the *ORF-3* mutation (blue), meaning that the duplex is further destabilized relative to the wild type sequence. Note that all shifts are localized to the same SIDD “well” as found in the wild type sequence. **Panel B.** Effect of *Prom-3* or *ORF-3* mutations on relative IS*5* insertion frequencies (*see text and Table 4*). Color coding is the same as in Panel A: black shows insertion frequencies for the wild type strain, red for the *Prom-3* (re-stabilized duplex) mutant, and blue for the *ORF-3* (further destabilized duplex) mutant.

If duplex destabilization allows frequent IS*5* insertion into a Py-T-A-Pu target site, one would anticipate that DNA sequence changes that decrease the G(x) values will lead to an increase in IS*5* insertion. To test for this possibility, we first analyzed the effect of *in silico* mutations in the first 15 codons of the *nfsB* gene on the G(x) values of the SIDD region. We found that introducing three third-position GC→AT base substitutions to replace three codons (3, 9 and 14) with synonymous codons had the effect of decreasing the G(x) values further (Fig 7A, blue plot line). We introduced these three changes into the chromosomal copy of the *nfsB* gene as described in *Materials and Methods*, and tested the effects on the appearance of total FZD^r^ mutants, total IS insertional mutants, and IS*5* insertional mutants. Our data (Table 4 and Fig 7B) show that decreasing the G(x) value (*ORF-3* mutant) had the effect of significantly increasing the fraction of *nfsB* mutants that had IS*5* insertions at the hotspot locus, while increasing the G(x) values (*PROM-3* mutant) had the opposite effect.

**Table 4:** Frequencies of total FZD^r^ (*nfsB*) mutants, total IS insertional mutants and IS5 insertional mutants.

Strain	FZD^r^ mutants per 10^8^ cells	Number of FZD^r^ mutants analyzed by PCR	All IS mutants (%) [*p*]^a^	IS*5* mutants (%) [*p*]
Wild type	71.8	126	58.7	50.8
PROM-3	61.1	118	37.2 [*<0*.*01*]	24.6 [*<0*.*005*]
ORF-3	99.1	118	88.1 [*<0*.*005*]	70.3 [*<0*.*01*]

^a^ “All IS mutants” denote the sum of IS1, IS2 and IS5 insertional mutants among FZD^r^ mutants analyzed by PCR. Significance was determined using the Chi-squared test.

### Potential evolutionary significance of IS*5* targeting to SIDD regions

Our preliminary analyses (not shown) of an extensive set of unselected IS*5* insertion sites in evolved cultures confirmed that while IS*5* hopping is favored at SIDD sequences, it is not exclusive to SIDD sites, indicating that other, as yet undiscovered DNA structural elements also modulate IS*5* insertion. Given the immense diversity of transposable elements, it is likely that a large variety of structural determinants will play roles in creating hot and cold spots for transposition by different elements. In the examples discussed in this communication, we have focused on known systems in which environmental stress conditions appeared to influence IS*5* insertion. We find that in all five cases, insertion events target tetranucleotide sequences embedded within SIDD sequences that are also enriched for H-NS binding.

A long-standing puzzle concerning the evolutionary forces that retain cryptic operons such as the *bgl* operon may be relevant to understanding how such IS*5* hotspots could have been selected. It has been speculated [65] that the *bgl* operon is retained for its potential benefit in utilizing aromatic β-glucosides encountered in the environment, but is nevertheless maintained in a cryptic form in a majority of cells because the same enzymes could also act on lethal substrates such as the environmentally-ubiquitous cyanogenic glucosides [66, 67]. The unstated presumption in this speculation is that mechanisms exist for reactivating, and then subsequently deactivating cryptic operons on an “as-needed” basis.

As discussed above, IS*5* can not only lead to gene activation by hopping into a hotspot within the control region of the gene, but it can also reverse the activation by precise excision from the control region, as demonstrated previously in the *fucAO*-*fucPIK* system [15]. We propose that some IS*5* hotspots evolved as a population-level regulatory mechanism for turning genes on and off in response to environmental stimuli. In addition to relatively non-specific nucleoid proteins such as H-NS that are required for transposition, locus-specific DNA-binding proteins such as GlpR and Crp may regulate transposon targeting in response to cognate environmental stimuli by masking or unmasking hotspots as discussed above for the *glpFK* model system. The ubiquity of transposable elements was long interpreted as evidence of a highly evolved and mutually beneficial relationship between transposable elements and the host genome, and this communication sheds light on one such relationship.

## Supporting information

S1 Table E. coli strains used in this study. (DOCX)

S2 Table Oligonucleotides used in this study. (DOCX)

## References

[1] Siguier P, Gourbeyre E, Chandler M. Bacterial insertion sequences: their genomic impact and diversity. FEMS Microbiol Rev. 2014;38(5):865–91. doi: 10.1111/1574-6976.12067 24499397 10.1111/1574-6976.12067 PMC7190074

[2] Aziz RK, Breitbart M, Edwards RA. Transposases are the most abundant, most ubiquitous genes in nature. Nucleic Acids Res. 2010;38(13):4207–17. doi: 10.1093/nar/gkq140 20215432 10.1093/nar/gkq140 PMC2910039

[3] Siguier P, Gourbeyre E, Varani A, Ton-Hoang B, Chandler M. Everyman's Guide to Bacterial Insertion Sequences. Microbiol Spectr. 2015;3(2):Mdna3-0030-2014. 10.1128/microbiolspec.MDNA3-0030-2014 26104715

[4] van der Woude MW, Baumler AJ. Phase and antigenic variation in bacteria. Clin Microbiol Rev. 2004;17(3):581–611, table of contents. doi: 10.1128/CMR.17.3.581-611.2004 15258095 10.1128/CMR.17.3.581-611.2004 PMC452554

[5] Bartlett DH, Silverman M. Nucleotide sequence of IS492, a novel insertion sequence causing variation in extracellular polysaccharide production in the marine bacterium Pseudomonas atlantica. J Bacteriol. 1989;171(3):1763–6. 2537827 10.1128/jb.171.3.1763-1766.1989 PMC209814

[6] Hammerschmidt S, Hilse R, van Putten JP, Gerardy-Schahn R, Unkmeir A, Frosch M. Modulation of cell surface sialic acid expression in Neisseria meningitidis via a transposable genetic element. EMBO J. 1996;15(1):192–8. 8598202 PMC449931

[7] Zhang Z, Saier MH Jr. A mechanism of transposon-mediated directed mutation. Mol Microbiol. 2009;74(1):29–43. doi: 10.1111/j.1365-2958.2009.06831.x 19682247 10.1111/j.1365-2958.2009.06831.x PMC2973706

[8] Zhang Z, Saier MH Jr. A novel mechanism of transposon-mediated gene activation. PLoS Genet. 2009;5(10):e1000689 doi: 10.1371/journal.pgen.1000689 19834539 10.1371/journal.pgen.1000689 PMC2753651

[9] Saier MH Jr., Zhang Z. Transposon-mediated directed mutation controlled by DNA binding proteins in Escherichia coli. Frontiers in microbiology. 2014;5:390 doi: 10.3389/fmicb.2014.00390 25136335 10.3389/fmicb.2014.00390 PMC4117983

[10] Wang X, Wood TK. IS5 inserts upstream of the master motility operon flhDC in a quasi-Lamarckian way. The ISME journal. 2011;5(9):1517–25. doi: 10.1038/ismej.2011.27 21390082 10.1038/ismej.2011.27 PMC3160685

[11] Zhang Z, Kukita C, Humayun MZ, Saier MH. Environment-directed activation of the Escherichia coli flhDC operon by transposons. Microbiology. 2017;163(4):554–69. doi: 10.1099/mic.0.000426 28100305 10.1099/mic.0.000426 PMC5775904

[12] Zhang Z, Kukita C, Humayun MZ, Saier MH Jr. Environment-directed Activation the Escherichia coli flhDC Operon by Transposons. Microbiology. 2017. 10.1099/mic.0.000426 PMC5775904 28100305

[13] Schnetz K, Rak B. IS5: a mobile enhancer of transcription in Escherichia coli. Proc Natl Acad Sci U S A. 1992;89(4):1244–8. 1311089 10.1073/pnas.89.4.1244 PMC48425

[14] Chen YM, Lu Z, Lin EC. Constitutive activation of the fucAO operon and silencing of the divergently transcribed fucPIK operon by an IS5 element in Escherichia coli mutants selected for growth on L-1,2-propanediol. J Bacteriol. 1989;171(11):6097–105. 2553671 10.1128/jb.171.11.6097-6105.1989 PMC210477

[15] Zhang Z, Yen MR, Saier MH Jr. Precise excision of IS5 from the intergenic region between the fucPIK and the fucAO operons and mutational control of fucPIK operon expression in Escherichia coli. J Bacteriol. 2010;192(7):2013–9. doi: 10.1128/JB.01085-09 20097855 10.1128/JB.01085-09 PMC2838058

[16] Vandecraen J, Monsieurs P, Mergeay M, Leys N, Aertsen A, Van Houdt R. Zinc-Induced Transposition of Insertion Sequence Elements Contributes to Increased Adaptability of Cupriavidus metallidurans. Frontiers in microbiology. 2016;7:359 doi: 10.3389/fmicb.2016.00359 27047473 10.3389/fmicb.2016.00359 PMC4803752

[17] Parker LL, Hall BG. Mechanisms of activation of the cryptic cel operon of Escherichia coli K12. Genetics. 1990;124(3):473–82. 2179048 10.1093/genetics/124.3.473 PMC1203941

[18] Hall BG, Xu L. Nucleotide sequence, function, activation, and evolution of the cryptic asc operon of Escherichia coli K12. Molecular biology and evolution. 1992;9(4):688–706. 1630307 10.1093/oxfordjournals.molbev.a040753

[19] Hall BG. Spectra of spontaneous growth-dependent and adaptive mutations at ebgR. J Bacteriol. 1999;181(4):1149–55. 9973340 10.1128/jb.181.4.1149-1155.1999 PMC93491

[20] Craig NL. Target site selection in transposition. Annu Rev Genet. 1997;66:437–74. 10.1146/annurev.biochem.66.1.437 9242914

[21] Manna D, Breier AM, Higgins NP. Microarray analysis of transposition targets in Escherichia coli: the impact of transcription. Proc Natl Acad Sci U S A. 2004;101(26):9780–5. doi: 10.1073/pnas.0400745101 15210965 10.1073/pnas.0400745101 PMC470751

[22] Fricker AD, Peters JE. Vulnerabilities on the lagging-strand template: opportunities for mobile elements. Annu Rev Genet. 2014;48:167–86. doi: 10.1146/annurev-genet-120213-092046 25195506 10.1146/annurev-genet-120213-092046

[23] Peters JE. Tn7. Microbiol Spectr. 2014;2(5). 10.1128/microbiolspec.MDNA3-0010-2014 26104363

[24] Choi J, Majima T. Conformational changes of non-B DNA. Chemical Society reviews. 2011;40(12):5893–909. doi: 10.1039/c1cs15153c 21901191 10.1039/c1cs15153c

[25] Du X, Wojtowicz D, Bowers AA, Levens D, Benham CJ, Przytycka TM. The genome-wide distribution of non-B DNA motifs is shaped by operon structure and suggests the transcriptional importance of non-B DNA structures in Escherichia coli. Nucleic Acids Res. 2013;41(12):5965–77. doi: 10.1093/nar/gkt308 23620297 10.1093/nar/gkt308 PMC3695496

[26] Benham CJ. Duplex destabilization in superhelical DNA is predicted to occur at specific transcriptional regulatory regions. J Mol Biol. 1996;255(3):425–34. doi: 10.1006/jmbi.1996.0035 8568887 10.1006/jmbi.1996.0035

[27] Zhabinskaya D, Madden S, Benham CJ. SIST: stress-induced structural transitions in superhelical DNA. Bioinformatics (Oxford, England). 2015;31(3):421–2. 10.1093/bioinformatics/btu657 25282644

[28] Benham CJ. Sites of predicted stress-induced DNA duplex destabilization occur preferentially at regulatory loci. Proc Natl Acad Sci U S A. 1993;90(7):2999–3003. 8385354 10.1073/pnas.90.7.2999 PMC46224

[29] Benham C, Kohwi-Shigematsu T, Bode J. Stress-induced duplex DNA destabilization in scaffold/matrix attachment regions. J Mol Biol. 1997;274(2):181–96. doi: 10.1006/jmbi.1997.1385 9398526 10.1006/jmbi.1997.1385

[30] Benham CJ, Bi C. The analysis of stress-induced duplex destabilization in long genomic DNA sequences. Journal of computational biology: a journal of computational molecular cell biology. 2004;11(4):519–43. 15579230 10.1089/cmb.2004.11.519

[31] Warming S, Costantino N, Court DL, Jenkins NA, Copeland NG. Simple and highly efficient BAC recombineering using galK selection. Nucleic Acids Res. 2005;33(4):e36 doi: 10.1093/nar/gni035 15731329 10.1093/nar/gni035 PMC549575

[32] Datsenko KA, Wanner BL. One-step inactivation of chromosomal genes in Escherichia coli K-12 using PCR products. Proc Natl Acad Sci U S A. 2000;97(12):6640–5. doi: 10.1073/pnas.120163297 10829079 10.1073/pnas.120163297 PMC18686

[33] Gilbert N, Allan J. Supercoiling in DNA and chromatin. Curr Opin Genet Dev. 2014;25:15–21. doi: 10.1016/j.gde.2013.10.013 24584092 10.1016/j.gde.2013.10.013 PMC4042020

[34] Bauer W, Vinograd J. The interaction of closed circular DNA with intercalative dyes. I. The superhelix density of SV40 DNA in the presence and absence of dye. J Mol Biol. 1968;33(1):141–71. 4296517 10.1016/0022-2836(68)90286-6

[35] Deweese JE, Osheroff MA, Osheroff N. DNA Topology and Topoisomerases: Teaching a "Knotty" Subject. Biochemistry and molecular biology education: a bimonthly publication of the International Union of Biochemistry and Molecular Biology. 2008;37(1):2–10. 10.1002/bmb.20244 PMC2643378 19225573

[36] Hatfield GW, Benham CJ. DNA topology-mediated control of global gene expression in Escherichia coli. Annu Rev Genet. 2002;36:175–203. doi: 10.1146/annurev.genet.36.032902.111815 12429691 10.1146/annurev.genet.36.032902.111815

[37] Wang H, Noordewier M, Benham CJ. Stress-induced DNA duplex destabilization (SIDD) in the E. coli genome: SIDD sites are closely associated with promoters. Genome research. 2004;14(8):1575–84. doi: 10.1101/gr.2080004 15289476 10.1101/gr.2080004 PMC509266

[38] Ak P, Benham CJ. Susceptibility to superhelically driven DNA duplex destabilization: a highly conserved property of yeast replication origins. PLoS computational biology. 2005;1(1):e7 doi: 10.1371/journal.pcbi.0010007 16103908 10.1371/journal.pcbi.0010007 PMC1183513

[39] Zhang Z, Saier MH Jr. Transposon-mediated activation of the Escherichia coli glpFK operon is inhibited by specific DNA-binding proteins: Implications for stress-induced transposition events. Mutat Res. 2016;793–794:22–31. doi: 10.1016/j.mrfmmm.2016.10.003 27810619 10.1016/j.mrfmmm.2016.10.003 PMC5136330

[40] Eze MO, McElhaney RN. The effect of alterations in the fluidity and phase state of the membrane lipids on the passive permeation and facilitated diffusion of glycerol in Escherichia coli. J Gen Microbiol. 1981;124(2):299–307. doi: 10.1099/00221287-124-2-299 7035612 10.1099/00221287-124-2-299

[41] Maisnier-Patin S, Roth JR. The Origin of Mutants Under Selection: How Natural Selection Mimics Mutagenesis (Adaptive Mutation). Cold Spring Harbor perspectives in biology. 2015;7(7):a018176 doi: 10.1101/cshperspect.a018176 26134316 10.1101/cshperspect.a018176 PMC4484973

[42] Andersson DI, Hughes D, Roth JR. The Origin of Mutants under Selection: Interactions of Mutation, Growth, and Selection. EcoSal Plus. 2011;4(2). 10.1128/ecosalplus.5.6.6 26442510

[43] Karlin S, Mrazek J, Campbell AM. Compositional biases of bacterial genomes and evolutionary implications. J Bacteriol. 1997;179(12):3899–913. 9190805 10.1128/jb.179.12.3899-3913.1997 PMC179198

[44] Harshey RM. Transposable Phage Mu. Microbiol Spectr. 2014;2(5). 10.1128/microbiolspec.MDNA3-0007-2014 PMC4486318 26104374

[45] Navarre WW. The Impact of Gene Silencing on Horizontal Gene Transfer and Bacterial Evolution. Advances in microbial physiology. 2016;69:157–86. doi: 10.1016/bs.ampbs.2016.07.004 27720010 10.1016/bs.ampbs.2016.07.004

[46] Navarre WW, Porwollik S, Wang Y, McClelland M, Rosen H, Libby SJ, et al Selective silencing of foreign DNA with low GC content by the H-NS protein in Salmonella. Science. 2006;313(5784):236–8. doi: 10.1126/science.1128794 16763111 10.1126/science.1128794

[47] Swingle B, O'Carroll M, Haniford D, Derbyshire KM. The effect of host-encoded nucleoid proteins on transposition: H-NS influences targeting of both IS903 and Tn10. Mol Microbiol. 2004;52(4):1055–67. doi: 10.1111/j.1365-2958.2004.04051.x 15130124 10.1111/j.1365-2958.2004.04051.x

[48] Peters JE, Craig NL. Tn7 recognizes transposition target structures associated with DNA replication using the DNA-binding protein TnsE. Genes Dev. 2001;15(6):737–47. doi: 10.1101/gad.870201 11274058 10.1101/gad.870201 PMC312648

[49] Hall BG. Activation of the bgl operon by adaptive mutation. Molecular biology and evolution. 1998;15(1):1–5. 9491599 10.1093/oxfordjournals.molbev.a025842

[50] Singh J, Mukerji M, Mahadevan S. Transcriptional activation of the Escherichia coli bgl operon: negative regulation by DNA structural elements near the promoter. Mol Microbiol. 1995;17(6):1085–92. 8594328 10.1111/j.1365-2958.1995.mmi_17061085.x

[51] Schnetz K. Silencing of Escherichia coli bgl promoter by flanking sequence elements. EMBO J. 1995;14(11):2545–50. 7781607 10.1002/j.1460-2075.1995.tb07252.x PMC398368

[52] Reynolds AE, Felton J, Wright A. Insertion of DNA activates the cryptic bgl operon in E. coli K12. Nature. 1981;293(5834):625–9. 6270569 10.1038/293625a0

[53] Venkatesh GR, Kembou Koungni FC, Paukner A, Stratmann T, Blissenbach B, Schnetz K. BglJ-RcsB heterodimers relieve repression of the Escherichia coli bgl operon by H-NS. J Bacteriol. 2010;192(24):6456–64. doi: 10.1128/JB.00807-10 20952573 10.1128/JB.00807-10 PMC3008536

[54] Fahrner KA, Berg HC. Mutations That Stimulate flhDC Expression in Escherichia coli K-12. J Bacteriol. 2015;197(19):3087–96. doi: 10.1128/JB.00455-15 26170415 10.1128/JB.00455-15 PMC4560290

[55] Soutourina O, Kolb A, Krin E, Laurent-Winter C, Rimsky S, Danchin A, et al Multiple control of flagellum biosynthesis in Escherichia coli: role of H-NS protein and the cyclic AMP-catabolite activator protein complex in transcription of the flhDC master operon. J Bacteriol. 1999;181(24):7500–8. 10601207 10.1128/jb.181.24.7500-7508.1999 PMC94207

[56] Lehnen D, Blumer C, Polen T, Wackwitz B, Wendisch VF, Unden G. LrhA as a new transcriptional key regulator of flagella, motility and chemotaxis genes in Escherichia coli. Mol Microbiol. 2002;45(2):521–32. 12123461 10.1046/j.1365-2958.2002.03032.x

[57] Yona-Nadler C, Umanski T, Aizawa S, Friedberg D, Rosenshine I. Integration host factor (IHF) mediates repression of flagella in enteropathogenic and enterohaemorrhagic Escherichia coli. Microbiology. 2003;149(Pt 4):877–84. doi: 10.1099/mic.0.25970-0 12686630 10.1099/mic.0.25970-0

[58] Krin E, Danchin A, Soutourina O. RcsB plays a central role in H-NS-dependent regulation of motility and acid stress resistance in Escherichia coli. Res Microbiol. 2010;161(5):363–71. doi: 10.1016/j.resmic.2010.04.002 20435136 10.1016/j.resmic.2010.04.002

[59] Wiebe H, Gurlebeck D, Gross J, Dreck K, Pannen D, Ewers C, et al YjjQ Represses Transcription of flhDC and Additional Loci in Escherichia coli. J Bacteriol. 2015;197(16):2713–20. doi: 10.1128/JB.00263-15 26078445 10.1128/JB.00263-15 PMC4507341

[60] Copeland MF, Weibel DB. Bacterial Swarming: A Model System for Studying Dynamic Self-assembly. Soft matter. 2009;5(6):1174–87. doi: 10.1039/B812146J 23926448 10.1039/B812146J PMC3733279

[61] Staib L, Fuchs TM. Regulation of fucose and 1,2-propanediol utilization by Salmonella enterica serovar Typhimurium. Frontiers in microbiology. 2015;6:1116 doi: 10.3389/fmicb.2015.01116 26528264 10.3389/fmicb.2015.01116 PMC4600919

[62] McCalla DR, Kaiser C, Green MH. Genetics of nitrofurazone resistance in Escherichia coli. J Bacteriol. 1978;133(1):10–6. 338576 10.1128/jb.133.1.10-16.1978 PMC221970

[63] Race PR, Lovering AL, Green RM, Ossor A, White SA, Searle PF, et al Structural and mechanistic studies of Escherichia coli nitroreductase with the antibiotic nitrofurazone. Reversed binding orientations in different redox states of the enzyme. J Biol Chem. 2005;280(14):13256–64. doi: 10.1074/jbc.M409652200 15684426 10.1074/jbc.M409652200

[64] Whiteway J, Koziarz P, Veall J, Sandhu N, Kumar P, Hoecher B, et al Oxygen-insensitive nitroreductases: analysis of the roles of nfsA and nfsB in development of resistance to 5-nitrofuran derivatives in Escherichia coli. J Bacteriol. 1998;180(21):5529–39. 9791100 10.1128/jb.180.21.5529-5539.1998 PMC107609

[65] Sankar TS, Neelakanta G, Sangal V, Plum G, Achtman M, Schnetz K. Fate of the H-NS-repressed bgl operon in evolution of Escherichia coli. PLoS Genet. 2009;5(3):e1000405 doi: 10.1371/journal.pgen.1000405 19266030 10.1371/journal.pgen.1000405 PMC2646131

[66] Conn EE. The metabolism of a natural product: lessons learned from cyanogenic glycosides. Planta medica. 1991;57(7 Suppl):S1–9. doi: 10.1055/s-2006-960222 17226216 10.1055/s-2006-960222

[67] Gleadow RM, Moller BL. Cyanogenic glycosides: synthesis, physiology, and phenotypic plasticity. Annual review of plant biology. 2014;65:155–85. doi: 10.1146/annurev-arplant-050213-040027 24579992 10.1146/annurev-arplant-050213-040027

