# Supplementary material for: Hopping into a hot seat: Role of DNA structural features on IS5-mediated gene activation and inactivation under stress
Source: PLoS One. 2017 Jun 30;12(6):e0180156. doi: 10.1371/journal.pone.0180156 (PMC5493358; doi:10.1371/journal.pone.0180156)
Supplement: S1 Table — (DOCX) [file pone.0180156.s001.docx]

**Table S1.** ***E. coli* strains used in this study**

| Strains | Genotype/relevant characteristics | | Source |
| --- | --- | --- | --- |
| BW25113 | Wild type, sensitive to FZD at 0.25 μg/ml | (32) | |
| BWstepI | IS5 insertion in *nfsA* promoter region, resistance to FZD at 1 μg/ml | This study | |
| BWK | Λ*galK* in BWstepI, Gal^-^ | This study | |
| PROM-3 | BWK with mutations that restabilize DNA duplex in the SIDD region of *nfsB* (decreased potential for DNA duplex melting) | This study | |
| ORF-3 | BWK with mutations that further destabilize DNA duplex in the SIDD region of *nfsB* (increased potential for DNA duplex melting) | This study | |
