# Supplementary material for: Hopping into a hot seat: Role of DNA structural features on IS5-mediated gene activation and inactivation under stress
Source: PLoS One. 2017 Jun 30;12(6):e0180156. doi: 10.1371/journal.pone.0180156 (PMC5493358; doi:10.1371/journal.pone.0180156)
Supplement: S2 Table — (DOCX) [file pone.0180156.s002.docx]

# Table S2. Oligonucleotides used in this study

| **Name** | **Sequence** | **Use** |
| --- | --- | --- |
| GalK-PROM3-P1 | catttttctcgcttaccatttctcgttgaaccttgtaatctgctggcacgcaacctgttgacaattaatcatcggca | Amplification of em7-*galK* for replacing “aattactt” in *nfsB* |
| GalK-PROM3-P2 | gaatgacgctttaaggcgacagaaatgatatccataaagactccatgtgatcagcactgtcctgctccttgtg | Amplification of em7-*galK* for replacing “aattactt” in *nfsB* |
| NfsB-R2 | tcaagcattttacgctcgttgaacac | Verification of em7-*galK* integration |
| GalK-sgP1 | gtaatctgctggcacgcaaaattactttcacatggagtctttatggatatcctgttgacaattaatcatcggca | Amplification of em7-*galK* for replacing “catttctgtcgccttaaagcgtcattccactaag” in *nfsB* |
| GalK-sgP2 | tttgatctgctcggcctgttccggggtaagttttttgctggcatcaaatgctcagcactgtcctgctccttgtg | Amplification of em7-*galK* for replacing “catttctgtcgccttaaagcgtcattccactaag” in *nfsB* |
| SIDD-wk | ctcgcttaccatttctcgttgaaccttgtaatctgctggcacgcaa**CC**ttact**G**tcacatggagtctttatggatatcatttctgtcgccttaaagcgtc | Three nucleotide substitutions (uppercase) rendering SIDD structure weaker |
| SIDD-wk-F | ctcgcttaccatttctcgttgaac | Amplification of SIDD-wk |
| SIDD-wk-R | gacgctttaaggcgacagaaatg | Amplification of SIDD-wk |
| SIDD-sg | caaaattactttcacatggagtctttatggatat**A**atttctgtcgccttaaa**A**cgtcattccactaa**A**gcatttgatgccagcaaaaaacttaccccg | Three nucleotide substitutions (capital) rendering SIDD structure stronger |
| SIDD-sg-F | caaaattactttcacatggagtc | Amplification of SIDD-sg |
| SIDD-sg-R | cggggtaagttttttgctggcatc | Amplification of SIDD-sg |
| NfsB-ver-F | aagaaatctccgaagcgttacttcg | Amplification of *nfsB* and its promoter region |
| NfsB-ver-R | agggttatgcaaatcaggagaatctg | Amplification of *nfsB* and its promoter region |
